# Gesture and Language Trajectories in Early Development: An Overview From the Autism Spectrum Disorder Perspective

**DOI:** 10.3389/fpsyg.2019.01211

**Published:** 2019-05-28

**Authors:** Sara Ramos-Cabo, Valentin Vulchanov, Mila Vulchanova

**Affiliations:** Language Acquisition and Language Processing Lab, Department of Language and Literature, Norwegian University of Science and Technology, Trondheim, Norway

**Keywords:** autism, non-verbal communication, verbal communication, gesture, developmental trajectory

## Abstract

The well-documented gesture-language relation in typical communicative development (TD) remains understudied in autism spectrum disorder (ASD). Research on early communication skills shows that gesture production is a strong predictor of language in TD, but little is known about the association between gestures and language in ASD. This review focuses on exploring this relation by addressing two topics: the reliability of gestures as predictor of language competences in ASD and the types of potential differences (quantitative, qualitative, or both) in the gesture-language trajectory in children on the autism spectrum compared to typically developing children. We find evidence that gesture production is indeed a reliable predictor of early communicative skills and that both quantitative and qualitative differences have been established in research in the development of verbal and non-verbal communication skills in ASD, with lower gesture rates at the quantitative level, and a trajectory that starts deviating from the TD trajectory only at some point after the first year of life.

## Introduction

One of the earliest signs of autism spectrum disorder (ASD) is the absence or the delayed onset of verbal and non-verbal communication behaviors. While there is a vast body of research showing that typically developing children’s (TD) early language is critically dependent on gesture production (Iverson and Goldin-Meadow, 2005; Rowe et al., 2008; Murillo and Belinchón, 2012), there are still many open questions concerning the role of gestures in the acquisition of linguistic abilities in children with ASD. This article offers an overview of the gesture-language relationship in ASD in an attempt to answer two key questions: (1) Are gestures a reliable predictor of language also in ASD? and (2) Are the potential differences in the trajectories of gesture and language development in ASD and TD infants quantitative (e.g., absence/lower level of gesture comprehension/production in ASD), qualitative (e.g., production of different gesture types and/or different hand configurations), or both?

## Gesture and Its Relation to Language Acquisition in Early Typical Development

A relationship between gesture and language acquisition in TD infants has been found systematically. In a meta-analysis where the gesture-language association was explored, Colonnesi et al. (2010) reported three main commonalities across 25 different studies. The first finding common to all the included studies was that both gesture comprehension and gesture production are involved in the subsequent acquisition of spoken language. The second main finding was that early gesture production onset correlated significantly with more advanced language competence later in development. Importantly, not all gestures appear to have the same predictive potential; deictic declarative gestures are specifically related to language acquisition.

Consistent with the first assertion, evidence shows that gestures can be identified not only as precursors but also as predictors of language, both at the lexical and syntactic level. In support of this, a number of studies report that infants first name objects that were previously referred to through gestures and that the transition from the one-word stage to the two-word stage in language acquisition is strongly supported by gesture-speech combinations (Capirci et al., 1996; Iverson and Goldin-Meadow, 2005; Iverson, 2010). Critically, not all gesture-speech combinations have the same predictive value. For instance, *complementary* combinations have the earliest onset and are aimed at reinforcing the semantic information provided verbally, in such a manner that the gesture and the utterance share referents (the object/location/event of interest). In contrast, *supplementary* combinations are complex combinations, where two different pieces of information mediated by different modalities (visual and motor in the case of gestures, and auditory for speech) are combined. Importantly, it is the emergence of supplementary combinations that predicts the production of the first two-word combinations in speech (Goldin-Meadow and Butcher, 2003). Thus, gesture-speech combinations mark the syntactic attainment of the child, but only supplementary combinations are linked to the development of more advanced grammar (syntax) (Iverson and Goldin-Meadow, 2005). Hence, the ability to integrate gestures and speech by conveying two different meanings (i.e., as in supplementary combinations) seems to be at the heart of the cognitive resources necessary to start producing two-word speech combinations.

In this vein, Özçalişkan and Goldin-Meadow (2005) showed that gesture-speech combinations continue to boost language acquisition throughout development after the stage when infants are able to produce two-word utterances. They tracked these combinations in TD toddlers at 14, 18, and 22 months showing that infants at 18 months produced constructions involving more than one argument by means of gesture-speech combinations (e.g., utter “mama” (=Argument 1), and point with the index finger to a chair to ask to be seated (=Argument 2) that later fully entered the expressive language domain (e.g., with both argument and verb expressed verbally “mama, sit”) at 22 months. In addition, gesture-speech combinations also appear to increase in complexity by the 22 months of age, with the emergence of constructions containing two predicates (e.g., “help me” produced as speech combined with a pointing gesture to a chair meaning “(intention to) sit” as the second predicate). Interestingly, once infants start producing two-word combinations in speech only, they do it in a way that mimics the rules of their mother tongue. That is, the two words are produced consistently with the word order of the infants’ L1. Thus, gesture development patterns, similarly to language acquisition, in a way that gestures first convey the meaning of single referents in the same manner words do, and afterwards, they broaden the semantic context in a sentence-like fashion with the emergence of gesture-speech combinations (Goldin-Meadow and Butcher, 2003). Thus, gesture-speech combinations mark the developmental point at which toddlers are about to expand their communication skills, by linguistic structures, such as combinations of arguments or predicates, emerging as gesture-speech combinations first and entering the verbal domain later in an increasingly more complex manner.

The second finding in the meta-analysis by Colonnesi et al. (2010) concerns the key role of the early emergence of gesture production in the growth of verbal abilities later on in development. Several studies have provided evidence in favor of the relation between gesture onset and the development of language comprehension and production on the one hand and gesture onset and higher gesture production rates (Butterworth and Morissette, 1996) on the other. Brooks and Meltzoff (2008) offer a potential explanation for the predictive power of early gesture over language outcomes. They argue that pointing has a bi-directional function; it aids infants by providing them with a communicative tool, and it makes caregivers prone to produce the labels that the toddler is pointing at, therefore, fostering the infant’s linguistic abilities.

Furthermore, the meta-analysis by Colonnesi and colleagues shows that not all gestures convey the same information, and only the ones carrying referential information have the potential to boost language attainment. Consistent with this, a recent study shows that deictic gestures are the only type of gestures that can predict later language development (Özçaliskan et al., 2016). Deictic gestures have the specific goal of directing the addressee’s attention toward a referent (the object/location/event of interest) and are context-dependent. That is, their meaning needs to be inferred through the information that the speaker (the one who draws the interlocutor’s attention) and the addressee are available in a shared-experience context (Tomasello, 2015), sometimes called “the common ground.” The inference involved in this process indicates the high cognitive demands that the comprehension of deictic gestures entails. It requires that children not only understand the referential intention of the speaker (i.e., the target of the message) but also grasp the social intention behind the deictic gesture (i.e., why is that referent important to the speaker) (Liebal et al., 2009). Thus, deictic gesture production constitutes a socially and cognitively demanding behavior. Deictic gestures come in the form of pointing, reaching, showing, and giving (see Iverson and Thal, 1998) and can be classified into two main categories from the point of view of their communicative motive: *imperative* and *declarative*. The imperative motive is the simpler communicative act, in which the speaker directs the addressee’s attention toward a desired or needed object (i.e., non-verbal requests). Conversely, the declarative motive is the most sophisticated one and refers to communicative instances where infants direct the addressees’ attention to something that they find interesting and need to communicate about.

It has been shown that each motive is related to two different gesture configurations: whole-hand pointing, in the case of imperatives and index finger pointing, in the case of the declarative motive, and even a manual preference of right-hand for declaratives. It has also been established that infants vocalize more frequently when producing index-finger pointing compared to whole-hand pointing (Cochet and Vauclair, 2010; Liszkowski and Tomasello, 2011). The main distinction, however, is based on the intention driving each specific gesture. While the motive of imperative gestures is to obtain something from the addressee, the main motivation for infants to use declarative gestures is to share experiences/thoughts/emotions about a referent, that is, declarative pointing is communicative in its nature. It is therefore no surprise that declarative pointing is so tightly yoked to language acquisition. Nonetheless, this type of gesture soon becomes obsolete, as it does not serve to explain in enough detail why a given referent is interesting; it is in this situation that language becomes necessary.

## Gesture-Language Trajectory in Typically Developing Infants

TD infants begin to point around 9–12 months of age (Carpenter et al., 1998). This gesture type emerges as an unintentional index-finger extension that follows a progressive increase in its complexity underpinned by the abilities of eye-gaze following and body-orientation shifting (Masataka, 2003). The first gestures that infants produce are *iconic* and *deictic*. Unlike *deictic* gestures, *iconic* gestures are non-context dependent and have intrinsic semantic meaning, as they depict one or more characteristics of the referent (McNeill, 1992). The progression toward deictic gestures starts by parents beginning to communicate with their children using pointing gestures, and then, infants at around 10 months of age start producing these same gestures (Clark and Sengul, 1978; Özçaliśkan and Dimitrova, 2013). However, between the 10th and 13th month of age, the most prevalent gestures are not *deictic*, but *give* and *ritualized requests*, which consist of repeated open/close hand movements. After the first birthday, infants have been shown to clearly use declarative pointing with the index finger (Esteve-Gibert and Prieto, 2014) and produce their first words. Later on, by the 16th month of age, they steadily produce gesture-speech combinations (Butterworth, 2003), and shortly after, at around 18 months, toddlers enter the two-word stage (Goldin-Meadow and Butcher, 2003) and begin to refine their linguistic skills.

These studies reveal that during the first stages of the development of communication skills, infants typically accomplish a series of key milestones to acquire ultimately the most complex form of human communication: speech. This trajectory, however, presents great variability even in neurotypical infants, with significant differences in the onset and development of the lexicon, gestures, and grammar, evidenced, among other sources, in the scores of the MacArthur-Bates Communicative Development Inventories (CDI) (Fenson et al., 1994). Word learning, specifically, has been shown to vary between genders, with a slight advantage for girls over boys (Fenson et al., 1994), as well as a diverse usage of word-learning strategies between monolinguals, bilinguals, and multilinguals (Byers-Heinlein and Werker, 2009).

These differences are even more salient in individuals with ASD, who show impairments in both, the verbal and the non-verbal domain of communication, with an even greater variability in that group. In the following sections, we present the differences in the ASD trajectory and discuss the nature of the variability.

## Are Gestures Reliable Predictors of Language in ASD?

Here, we seek to establish whether gesture-language relation is present in ASD, and by characterizing the core differences of ASD gesture development, we seek to ascertain whether those differences are primarily quantitative or qualitative.

### Gesture and Language Correlation in ASD

Several studies have shown that despite a delay in the onset of gesture and language production (Charman et al., 2003; Mitchell et al., 2006), the relation between gesture and language seems to remain significant in children on the spectrum (Ingersoll and Lalonde, 2010). A recent study supports the idea that the gesture-language relation is present also in infants with ASD (Özçalışkan et al., 2017). In a longitudinal study, the authors recorded mother-child interactions of ASD, Down’s syndrome (DS), and TD children in a semi-naturalistic play protocol that elicited *requesting* and *commenting* interactions. This study found that the objects referred to gesturally entered the speech domain later on in both TD and ASD children. Consistent with these findings, (Tager-Flusberg et al., 1990) found that, later on, ASD children also follow the same developmental trajectory as their TD peers in the oral language domain. The measures collected (mean length of utterance, index of productive syntax, lexical diversity, and word class distribution) bimonthly in a 12- to 26-month period during mother-child interactions showed similarities in the acquisition of lexicon and grammar in both groups, suggesting adequate development of structural language.

Özçaliskan et al. (2016) also explored the gesture-language relation in ASD by tracking the gesture production and subsequent language outcomes of 18-month-old TD (*N* = 23) and 30-month-old ASD children (*N* = 23). They explored which type of gestures (*deictic*, *give*, *iconic,* or *conventional*) and which communicative function (*commenting* or *requesting*) better predicted later language development in this population. To this end, they used a semi-naturalistic interactive task with two communicative contexts (eliciting commenting and request-making) and assessed infants’ vocabulary size 1 year later. They found that only deictic gestures predicted vocabulary in both TD and ASD children, but with a significantly lower prevalence (70% ASD vs. 96% TD) and frequency (45% ASD vs. 60% TD) of these gestures in the ASD group. Importantly, while TD infants showed a clear tendency to produce more deictic gestures in the commenting context than in the requesting context, the ASD group did not show a difference in the gesture types produced across contexts. Similarly, Ökcün-Akçamuş et al. (2017) found that declarative deictic gestures together with conventional/pantomime gestures predicted vocabulary outcomes (i.e., number of words) in children with ASD in a 3- to 8-year-old age range^1^, while imperative deictic gestures did not. Additional findings showing that infants with language delay parallel ASD children on decreased rates of gesture production support the connection between gesture deficits and language difficulties, with the difference that such reduced gesture production seems to hinder both verbal and non-verbal communicative development over a longer period in individuals with ASD compared to the language delay group (Attwood et al., 1988; Lebarton and Iverson, 2016). Thus, evidence suggests that in spite of the delay in the emergence of gestures, the linguistic trajectory goes hand in hand with the development of gesture comprehension and gesture production also in ASD children. It is nevertheless worth noting that the fact that gestures and language are highly correlated does not imply that there is a causal relation between the two, rather the relation is bidirectional in a way that gesture and language mutually influence each other (Kelly et al., 2010) and constitute two dimensions of an integrated system used for communication (McNeill, 1992; Bernardis and Gentilucci, 2006). More importantly, according to the evidence presented above, this notion of a unified non-verbal and verbal communication system would be also applicable to ASD, which suggests that gestures might be reliable predictors of language in autism.

### Gesture-Language Trajectory in Children With ASD

Despite the resemblance in the structure of the developmental paths of TD and ASD, it is apparent that ASD infants and children present with poorer verbal and non-verbal communicative skills than their TD peers. There is emerging evidence showing that social-communication skills in ASD do not differ significantly from TD during the first year of life, with identical outcomes for TD and high-risk infants who go on to receive an autism diagnosis. This is evidenced on measures based on parental reports on social engagement and quality of interactions, as well as on objective measures, such as social orienting and responsiveness (e.g., gaze to face vs. objects) (Rogers, 2009; Elsabbagh and Johnson, 2016). This trajectory, nevertheless, begins to diverge soon after, with a steady decline in the growth rates of both gesture and language production (Iverson et al., 2017) and a decline in social engagement in ASD toddlers. This may reflect a bifurcated developmental trajectory where ASD and TD share the same starting point and then continue with differentially shaped paths, with autism emerging as a behavioral decline (regression) in social engagement, both visual and vocal, in the second year of life (Bosl et al., 2018). More longitudinal comparative studies on the ASD and TD trajectories are needed to test out the presence of a developmental trajectory that commences at the same start point and eventually (around the 12th to 24th month of life) deviates in the case of ASD.

A large set of studies attempting to identify the origin of these different developmental paths indicate mainly quantitative differences in the communication trajectory between ASD and TD children by showing overall lower gesture rates in ASD compared to TD (Mundy et al., 1986; Özçaliskan et al., 2016). Attwood et al. (1988) reported that this lower gesture rate is also found in ASD adolescents and that this is a pattern present in all individuals from the lowest to the highest end of the spectrum, irrespective of their intellectual abilities. Thus, the decreased number of gestures produced can only be accounted for in terms of the autistic symptomatology and not by the variability in the level of functioning or in cognitive abilities of individuals with ASD. The deficits in gesture production are also evident in studies with infants at high risk for developing autism (HR). These studies allow for following up the development of infants before the disorder can be diagnosed and, as such, are extremely valuable to detect subtle changes in early development that might provide behavioral markers of ASD. In a study with a HR sample, Iverson et al. (2009) tested the communicative behaviors (vocalizations, gestures, gesture+speech combinations) of HR and TD toddlers at 13 and 18 months of age. They found the lowest rates of communicative behaviors in the HR toddler group who were later diagnosed with ASD at both ages. Interestingly, a significantly lower communicative rate was also found between the HR infants who did not go on to develop ASD and their TD peers. Similarly, Lebarton and Iverson (2016) monitored the gesture production of a group of 2-year-old HR children for 1 year. The results showed different trajectories for the HR children who were diagnosed with ASD or language delay (LD) and for the children who did not receive a diagnosis. The ASD and LD children showed significantly lower gesture rates than the children with no diagnosis, with *pointing gestures* being the most markedly affected. Notably, this reduced gesture frequency was also present in 3-year-olds only in the ASD group.

According to a different view, however, the differences between gesture production in ASD and typically developing children are mainly qualitative. Supporting this view, some studies have found evidence suggesting that the gesture-language trajectory in itself is different in children with ASD. For instance, in a seminal study on qualitative gesture alterations in ASD, Baron-Cohen (1989) investigated the presence of impairments in the production and comprehension of proto-imperative and proto-declarative pointing in children with and without ASD ranging from 6 to 16 (ASD) and 3 to 5 (TD) years of age, as well as a sample of children with Down syndrome (DS). Imperative pointing comprehension was tested with the experimenter facing a set of toys close to the child, but out of reach for the experimenter. The experimenter then pointed at one of the four toys and asked what he/she was trying to say with that gesture. Children’s responses were then scored as pass, if children showed with their actions or verbal responses that they had understood the communicative intention of the experimenter or fail if they did not. The comprehension of declarative pointing was assessed with the experimenter pointing and shifting his/her eye-gaze into one of the three locations of interest that the child could not see, then looking back at the child, and then asking the same question as in the previous condition. Pass/fail response assessment was also done in accordance with the actions or responses of the participants. Results showed no group differences in proto-imperative pointing; however, the ASD children performed much more poorly in the comprehension of declarative pointing task compared to the controls. Remarkably, most of the ASD individuals in the sample (14/20) did not respond in any way, when the experimenter produced the declarative pointing. The same study assessed pointing production in three groups of 10 children each. One group of children with ASD in the age range of 2 to 5, another group of TD children between 1 and 2 years of age, and a group of intellectually disabled children ranging 3 to 6 years of age. Children were videotaped during play while accompanied by two female workers. Results at the production level reflected those of the comprehension domain, as there were no differences in the proto-imperative pointing production between the three groups, but a significant difference was found in the production of proto-declarative gestures, with ASD children producing fewer declarative pointing gestures.

Goodhart and Baron-Cohen (1993) further investigated declarative gesture production in ASD. On the premise that pointing can also be used with non-social purposes, they investigated whether the production of *referential* pointing was impaired in ASD. The authors defined this type of pointing as “pointing to name or identify one object as distinct from another” (p. 227) and specified that it can be used to help direct one’s own focus of attention. They assessed the production of spontaneous referential and declarative pointing in 7- to 18-year-old ASD participants matched on verbal age to a control group of TD children between 1 and 7 years of age, with a procedure where children were given a book to look at. This methodology allowed for making a clear dissociation between social and non-socially loaded pointing gestures for the first time, by creating an environment where the children could choose to establish eye-contact and try to engage in conversation with the experimenter or not. Findings showed that referential pointing is spared in ASD, indicating that it is not the production of declarative pointing in its broad sense that is affected, but the social interaction that the act of pointing declaratively carries. This suggests that the capacity of directing the attention of the addressee toward a given referent for the mere fact of initiating or engaging in a social interaction is severely affected in ASD. This fact, in turn, might account for the delayed/impaired language acquisition in ASD. Furthermore, language emergence and development strongly depends on *declarative* gestures, a type of gesture that is mostly present in caregiver-child interactions with social communicative motive.

A series of studies have been reporting both quantitative and qualitative differences between ASD and TD communication development. A recent study (Iverson et al., 2017) reported quantitative and qualitative differences in early vocabulary and gesture development in ASD and TD infants. With a set of four samples (a HR group that went on to receive an ASD diagnosis, a language delayed HR group, a HR with no other diagnosis, and a control group with no ASD risk), they tracked the development of communication skills in participating infants in their first 2 years of life. The MacArthur-Bates Communicative Development Inventory (CDI) was collected monthly (from the 8th to the 14th month) with the information provided by the infants’ parents or caregivers. Results showed, on the one hand, quantitative differences, by evidencing the slowest growth rate in the HR that eventually received an ASD diagnosis (HR-ASD). On the other hand, and more importantly, qualitative differences were found in the trajectories of the HR-ASD and in the HR infants who showed a delay in language (HR-LD) compared to the control group at low risk for ASD. It is worth mentioning that the CDI scores on early and later gestures and word comprehension and word production growth showed an identical trajectory for the HR-ASD and the control group at 8 months. This pattern, however, displayed a disruption at 11 months that persisted for the rest of the investigated age range (i.e., 24 months). It is noteworthy that the HR-LD infants obtained the lowest scores of all groups at 8 months in gesture and word comprehension, but their development accelerated eventually to reach the same developmental stage as controls by the 14th month. Similarly, a longitudinal study describing the different trajectories of HR infants from 9 to 24 months (Franchini et al., 2018) has shown different trajectories for TD, HR-TD (i.e., infants at risk with no later diagnosis), HR-ASD, and HR-ASD-LD infants. As expected, the HR infants who later went on to receive a diagnosis showed slower verbal and non-verbal communication trajectory compared to the TD and HR-TD groups, and importantly, the HR-ASD-LD (high-risk infants who developed ASD associated with language delay) had the slowest trajectory of the three groups.

Furthermore, Colgan et al. (2006) found that children on the spectrum do not only display lower gesture rates but a lower range of gesture types too, exhibiting a less diverse gesture production. In a retrospective study of gesture emergence in ASD, Colgan et al. (2006) collected home videotapes of ASD and TD children and analyzed gesture production during the social interactions of ASD and TD children from the 9th to the 12th month of age obtaining results that showed an association between the decreased variety of gesture use and ASD diagnosis.

Consistent with previous studies documenting a lower rate of pointing gestures in ASD compared to TD, Mastrogiuseppe et al. (2015) also showed that Down syndrome (DS) children outperform ASD in the total number of gestures produced, evidence that supports the assumption that gesture impairment is autism-specific. More importantly, this study was devoted to describing the specific features that characterize ASD gestures. To that end, the authors ran a comprehensive cross-sectional study exploring gesture use in naturalistic mother-child play interactions where gesture data were coded and analyzed to describe the differences between TD, DS, and ASD toddlers in the age range of 16–31, 29–48, and 30–60 months, respectively. The study documents five qualitative variances in the gestural behavior of ASD infants of high relevance: (1) the ASD group demonstrated the lowest rate of *conventional-interactive* gestures (arbitrary gestures whose meaning is culturally bound), (2) ASD children showed the lowest rate of *pointing* gestures, (3) the ASD group had the highest proportion of *ritualized requests*, and (4) the children with ASD were the only ones to produce *instrumental* gestures (consisting of taking the hand or the arm of the addressee to indicate him/her to take a specific action). Results showing a decreased amount of socially loaded gestures such as *conventional-interactive* or *pointing* gestures fit in with previous findings showing a marked impairment in *deictic declarative* gestures (Baron-Cohen, 1989; Goodhart et al., 1993); likewise, the highest proportion of *ritualized requests* in ASD goes in line with spared imperative gesture in this population. Finally, the presence of *instrumental* gestures only in the ASD group is of utmost importance, as it pinpoints a potential ASD-specific sign. Given the paramount importance of these gestures, as a potential behavioral marker of the condition, it is striking to find that, to our knowledge, only one other study has reported *instrumental* gestures in ASD (Stone et al., 1997). That study reported the presence of *instrumental* gestures in the context of a commenting and requesting elicitation study with 2- and 3-year-old ASD children, who used these types of gestures with the experimenter.

## Conclusions and Future Directions

This review explored, on the one hand, whether gestures can reliably predict language in ASD and, on the other, whether the divergence in the verbal and non-verbal communication trajectory of individuals on the spectrum is due to quantitative or qualitative differences when compared to neurotypical children. Two main conclusions can be drawn from the findings of the reported studies; firstly, the gesture-language association is also present in ASD, and secondly, the differences in the development of communication skills in ASD are both quantitative and qualitative.

Regarding the first conclusion, multiple studies have shown that gestures can predict language abilities in ASD (Tager-Flusberg et al., 1990; Ingersoll and Lalonde, 2010; Özçalışkan et al., 2017) and, in particular, *deictic declarative* gestures, which have proven as a reliable language predictor in both TD and ASD infants (Özçaliskan et al., 2016). This type of gesture has also been identified as the most markedly impaired in ASD (Baron-Cohen, 1989; Goodhart and Baron-Cohen, 1993; Mastrogiuseppe et al., 2015), a finding consistent with the language delay/impairment which can be observed early in that group of children. The fact that these types of gestures place higher social and interactive demands might be what underlies the impairment (Goodhart and Baron-Cohen, 1993) and could therefore constitute a powerful tool for early interventions. Previous studies show that gesture intervention improves vocabulary in TD children (LeBarton et al., 2015), which might indicate that the inclusion of *deictic declarative gestures* in early ASD intervention programs could be extremely beneficial. Reinforcing the production of these types of gestures may provide these children with an important means of non-verbal communication that can, at the same time, expand verbal abilities and, potentially, translate in the improvement of social interaction skills of children with ASD.

Taken together, the studies investigating the trajectory of non-verbal communicative skills in ASD indicate the presence of both quantitative and qualitative differences when comparing to the neurotypical development path. A recurrent quantitative difference in the literature is the lower gesture rate among ASD samples compared to TD samples (Mundy et al., 1986; Attwood et al., 1988; Colgan et al., 2006; Mastrogiuseppe et al., 2015; Lebarton and Iverson, 2016; Özçaliskan et al., 2016) and samples with other developmental deficits (Mastrogiuseppe et al., 2015). With regard to the qualitative differences, multiple differences have been reported, such as the impairment in the domain of *deictic declarative* gestures (Baron-Cohen, 1989; Goodhart and Baron-Cohen, 1993) or the uniqueness of *instrumental* gestures in ASD (Mastrogiuseppe et al., 2015).

Interestingly, some studies have reported evidence of quantitative and qualitative differences simultaneously (Colgan et al., 2006; Mastrogiuseppe et al., 2015; Iverson et al., 2017). This pattern of quantitative and qualitative differences is present from infancy (Mundy et al., 1986; Colgan et al., 2006; Özçaliskan et al., 2016) and persists throughout childhood (Baron-Cohen, 1989), through to adolescence (Attwood et al., 1988). However, the delays in the development of verbal and non-verbal communication do not surface until the first year of life of infants who go on to receive an ASD diagnosis (Iverson et al., 2017), providing a potential explanation to why some studies have failed to find any differences between the early communication skills in ASD and TD groups. This is, perhaps, one of the most important findings of the last decade in the field, as it shows that, in early infancy, infants on the spectrum may develop communication abilities similarly to TD, but the developmental trajectory changes abruptly between the first and second year of life (Rogers, 2009; Iverson et al., 2017). This pattern with a seemingly intact early development, which suffers a sudden decline at some point between the first 2 years of life, is not limited to the communication skills domain, but has also been found in social, cognitive, and adaptive behaviors (Ozonoff et al., 2010; Bussu et al., 2018). At any rate, caution is warranted when interpreting these findings, as much more supporting evidence is needed before it can be concluded that regression is inherently tied to ASD, especially given the great heterogeneity of the condition. In this regard, new research focusing on the early bio-/neuro-markers of the condition (Bosl et al., 2018), rather than reliance on overt behavior, could move the field further and potentially solve the question of the presence of regression in ASD by providing the means to identify alterations – or their absence – in early infancy. This is especially relevant, given that such changes are hardly detectable by most current social-communication behavior measures in young infants.

In her review, Rogers (2009) highlighted that there are no behavioral markers of ASD before 6 months of age and stressed the relevance of the appearance of the so-called secondary symptoms simultaneously or even before the delays or impairments in social and communication abilities. This leads to the conclusion that ASD cannot be characterized as a predominantly social-communication disorder. Another important question she pointed out is the extraordinary variety in the course of symptom presentation and in symptom severity for each specific symptom and each specific child with ASD. This remarkable inconsistency across the onset of ASD symptoms has been evidenced by the cases of HR infants who did not meet the criteria for ASD when followed up and assessed at early ages, but who presented with ASD symptoms later at pre-school or school (Davidovitch et al., 2015; Brian et al., 2016; Ozonoff et al., 2018). Although the symptomatology and its timing vary from case to case, and while this supports the argument that ASD is not a social and communication disorder primarily, the role of communication impairments, specifically that of gestures, is unquestionable. All the non-verbal communication studies in the review showed gesture impairments in all the HR-ASD samples in toddlers above 12 months of age (Rogers, 2009), which shows that gesture impairment, unlike many other signs that are found in some individuals on the spectrum but not in others, is pervasive in ASD. This fact reinforces the idea that gestures could be eligible for becoming proxy indicators of ASD, especially when the initial symptoms may be so diverse across the spectrum.

The study of gesture in ASD is therefore of crucial importance, particularly in the absence of studies investigating gesture hand configurations in ASD. It is well established that TD children use mainly extended index finger when pointing declaratively, possibly, the most critical gesture type in the study of the gesture-language relationship in infant and child development. The study of hand configurations could contribute crucial knowledge of gesture function and gesture types and, conceivably, help identify differential or alternative communication behaviors in ASD, a goal with important implications in clinical work, diagnosis, and the development of interventions. In this sense, the study of *instrumental* gestures seems immensely promising and, yet, remains understudied.

Another aspect of communication that has not received much attention in the study of autism is gesture-speech combinations. This is quite surprising considering that research on gesture-speech combinations has the potential to shed light on the disruptions in the verbal and non-verbal communication trajectory and add up to the knowledge of word learning in ASD. In their recent review, Manwaring et al. (2018) pointed out that only a single study investigating the gesture-language combinations in HR infants (Winder et al., 2013) has been carried out. Winder et al. (2013) conducted a longitudinal study with follow-up of HR infants at 13 and 18 months and showed fewer *show* and *pointing* gestures, non-words, words, and gesture+non-words and gesture+speech combinations in two tasks including an unstructured play protocol and a semi-structured play protocol with caregivers. The results showed that the HR-ASD infants were at the bottom or nearly at the bottom of the distribution of all the explored communicative instances (i.e., *show* and *pointing* gestures, non-words, words, gesture+non-words combinations, and gesture+speech combinations). More importantly, even though only 3 out of the total 15 HR participants went on to receive an ASD diagnosis later on, there was a significant difference between the verbal and non-verbal abilities of the HR and the control group. This raises the question of the necessity to study the developmental trajectory of HR infants who do not develop ASD (HR-noASD). Thus, a recent study showed an increased prevalence of language delay in HR-noASD (Marrus et al., 2018). In that study, the authors performed a meta-analysis of standardized language scores and diagnostic outcomes from nine HR studies, along with analyzing the same measures in a cohort study of 133 HR-noASD and 69 TD infants. Given that both analyses resulted in HR-noASD samples showing significantly lower language scores, Marrus et al. (2018) suggest language delay as a possible endophenotype for ASD. This research line could certainly provide exceptionally valuable information to widen the notion of the ASD broad phenotype that could ultimately increase our understanding of the spectrum.

Similarly, the findings of Iverson et al. (2017) and Franchini et al. (2018) showing differences in the gesture trajectories of HR infants who did not receive an ASD diagnosis and HR infants who went on to develop ASD mirror previous studies distinguishing late-talking infants with subsequent typical development from infants with persisting language delay (Thal and Tobias, 1992). In both cases, the early stages of infants from no diagnosis groups (the HR infants who were not diagnosed with ASD and the late-talkers who were not diagnosed with language impairment) were marked by decreased gesture production that eventually caught up with age-expected gesture rates. In this sense, the study of the differences between the HR infants who initially present with difficulties, but finally follow the typical trajectory, and the infants who end up developing ASD would be critical. This could allow describing the mechanisms that enable infants to catch up with their TD peers and mechanisms that conspire to disrupt the developmental trajectory and lead to acceleration in the severity of the symptoms.

Finally, the necessity for methods that will ensure their replicability needs mention. Despite the ecological validity of methods, such as unstructured interaction observations and/or analyses of home-video recordings, the use of these methods makes controlling for potential confounding factors extremely difficult, as well as making subsequent analyses subject to biases. We advocate gesture-language elicitation methodologies that can allow for naturalistic caregiver-child interactions in controlled settings (e.g., research paradigm employed in Linda Smith’s group, Smith and Gasser, 2005) that will make the quality of research on communication skills in infancy and in ASD, in particular, improve substantially.

## Author Contributions

SR-C conducted the review and prepared the manuscript. MV and VV edited the manuscript for submission.

### Conflict of Interest Statement

The authors declare that the research was conducted in the absence of any commercial or financial relationships that could be construed as a potential conflict of interest.

## Footnotes

^1^Given the young age of the participants in this study, it is worth noting that most of the studies reported in the present review included a sample of children who had already been diagnosed with ASD by an external clinician and had their diagnosis verified by the researchers. This diagnostic confirmatory assessment is typically done either by assessing the child with the Autism Diagnostic Observation Schedule—Generic (ADOS–G; Lord et al., 2000) or by using parental interview protocols, such as the Autism Diagnostic Interview-Revised (ADI-R; Lord et al., 1994) or the Childhood Autism Rating Scale (CARS; Schopler et al., 1980, 2010). In this sense, the ADOS and CARS (Kleinman et al., 2008; Guthrie et al., 2013) have shown to have the highest diagnostic stability when diagnosing young children.
